# Ecology and genetic structure of the invasive spotted lanternfly *Lycorma delicatula* in Japan where its distribution is slowly expanding

**DOI:** 10.1038/s41598-022-05541-z

**Published:** 2022-02-01

**Authors:** Ayano Nakashita, Yayun Wang, Sihan Lu, Keisuke Shimada, Tsutomu Tsuchida

**Affiliations:** 1 School of Science, University of Toyama, Toyama City, Toyama 930-8555 Japan; 2 College of Protection, Key Laboratory for Biology and Sustainable Management of Plant Diseases and Pest of Anhui Higher Education Institutes, Anhui Agricultural University, Hefei, 230036 China; 3 Ishikawa Museum of Natural History, Ri-441, Choshi-machi, Kanazawa City, Ishikawa 920-1147 Japan; 4 Faculty of Science, Academic Assembly, University of Toyama, 3190 Gofuku, Toyama City, Toyama 930-8555 Japan

**Keywords:** Entomology, Invasive species

## Abstract

*Lycorma delicatula* has expanded its distribution from China to Japan, Korea, and the USA, causing significant economic damage to vineyards in the latter two countries. However, in Japan, *L. delicatula* has long been limited to the Hokuriku region, central Japan, and no significant damage to crops has been reported since it was first reported there in 2009. Manipulation experiments and field observations in the Hokuriku region, where winter precipitation is extremely high, revealed that egg numbers and hatchability were significantly reduced in exposed places, especially when wax was excluded from the egg mass. Phylogenetic analysis showed that the population in Japan could be divided into at least two groups. Most *L. delicatula* samples from Hokuriku formed a clade with those from northwestern China. Samples from Okayama, where the distribution of *L. delicatula* was recently confirmed, had the same haplotype as those from central China, Korea, and the USA. These results suggest that environmental factors and genetic characteristics of *L. delicatula* are involved in the relatively slow expansion of its distribution in Hokuriku. Conversely, in Okayama, where precipitation is relatively low, the rapidly increasing haplotype in Korea and the USA was detected, leading to concerns that its distribution will expand further.

## Introduction

The spotted lanternfly, *Lycorma delicatula* (White), is a sap-feeding insect native to China. The main host plant of this species is the tree of heaven, *Ailanthus altissima*^1,2^, but it can utilise more than 70 plant species such as apples and grapes^3–5^. *L. delicatula* damages or even kills trees, including crops, by feeding on large amounts of plant sap and excreting honeydew on the leaf surfaces which promotes the growth of sooty mould fungus on them^3,4^.

Recently, *L. delicatula* has expanded its distribution and has invaded several other countries. Since its flight ability is poor, invasion of *L. delicatula* seems to occur through the transport of plants or other materials to which egg masses are attached^6,7^. In South Korea, an outbreak was reported in 2004. Thereafter, its distribution expanded throughout South Korea by 2011^4,8^. In the USA, *L. delicatula* was first identified in Pennsylvania in 2014^9^ and then its distribution expanded and its density increased, currently occupying 12 neighbouring states^4,10^ (Fig. S1c). In both South Korea and the USA, *L. delicatula* has caused significant economic damage to vineyards^4,11,12^. Many European countries are considered to be at high risk of invasion by *L. delicatula* through the global wine trade^13^. Hence, effective control strategies against *L. delicatula* are urgently needed.

In Japan, *L. delicatula* was first reported in Komatsu City, Ishikawa Prefecture in 2009 (site no. 7 in Fig. S1b)^14^. In 2013, *L. delicatula* was found in the neighbouring prefecture, Fukui, (site no. 9 in Fig. S1b)^15,16^ after which its distribution has gradually been expanding. In 2017, *L. delicatula* was found in Osaka Prefecture^17^ and in Okayama Prefecture in 2019 (site no. 10 in Fig. S1a)^18^. Therefore, in Japan, the distribution of *L. delicatula* has long been limited to the central parts of the country, the Hokuriku region (Fig. S1b), since it was first reported. Currently, the distribution of *L. delicatula* is sporadic, although the preferred host plant, *A. altissima*, is widely distributed throughout Japan^19,20^. To date there have been no reports of significant damage to agricultural crops caused by *L. delicatula,* suggesting that some environmental and/or genetic factors have prevented its distributional range expansion in Japan. Towards identification of these factors, the ecology and genetic structure of *L. delicatula* in Japan should be examined.

In this study, we conducted an ecological survey of *L. delicatula* in Kanazawa City, Ishikawa Prefecture, Japan. We observed seasonal variations in emergence, mating, and oviposition behaviour. We also evaluated egg survival under different conditions (sheltered or exposed egg deposit sites), with or without waxy deposits, on egg masses. Moreover, to evaluate the genetic structure of *L. delicatula* in Japan, molecular phylogenetic analysis was performed using newly collected samples from eight sites in Japan and one site in China and compared them with previously obtained sequences from China, South Korea, Japan, and the USA^11,21^.

## Results

### Seasonal occurrence of developmental stages of *L. delicatula*

**Figure 1:**
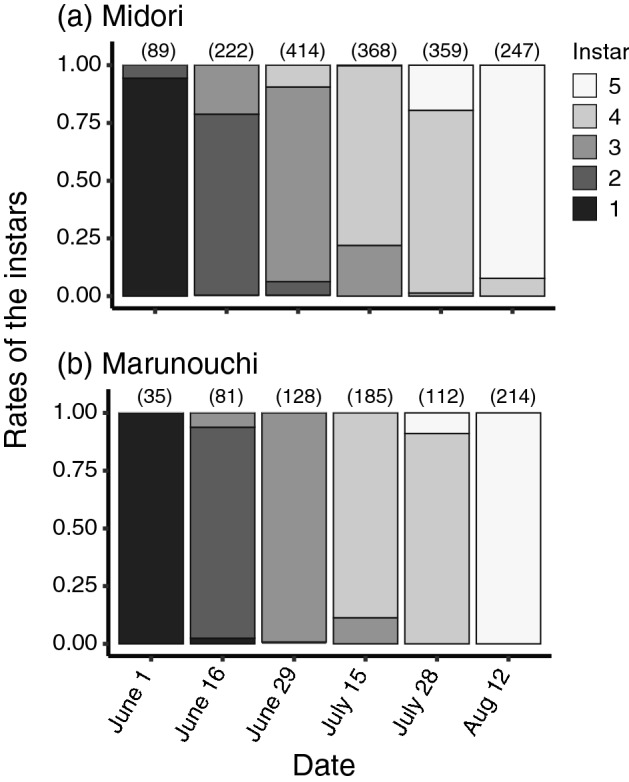
Seasonal occurrence of developmental stages of *Lycorma delicatula* at Midori (**a**) and Marunouchi (**b**) in Kanazawa City. Sample size is provided in parentheses.

The 1st instar larvae were found on June 1st at two study sites, Midori and Marunouchi, in Kanazawa city (Fig. 1). After that, the age structures of the populations changed approximately every two weeks until 15 July; the previously observed instar became infrequent, and the next older instar became predominant. The 4th instar larvae were mainly observed on 15 July and 28 July in the two sites. The first adult (5th instar) was observed on 15 July in Midori (Fig. 1a), and subsequently adults began to be observed at both sites from 28 July onwards (Fig. 1a,b).

**Figure 2:**
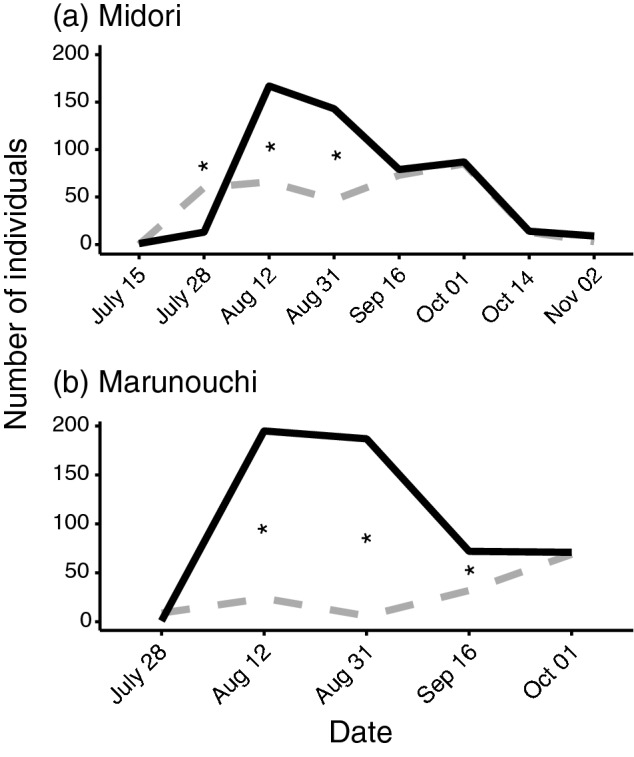
Number of female and male adults collected on each date at Midori (**a**) and Marunouchi (**b**). Black solid line indicates female, and grey dashed line indicates male. Asterisk indicates that the proportion of adult *Lycorma delicatula* significantly differed from the 1:1 sex ratio (P < 0.001, two-sided binomial test with Bonferroni correction).

There were significant differences in the numbers of adult males and females captured at several dates (Fig. 2). The number of adult females increased after their first emergence, reached a peak on 12 August, and then declined. There were fewer adult males than females at both sites. In Midori, a larger number of males were captured on 28 July; but thereafter, the number of males captured was approximately half that of females until 31 August (Fig. 2a). From 16 September, adult males and females were captured in equal proportions in Midori. A similar tendency was observed in Marunouchi (Fig. 2b).

### Mating and oviposition behaviour of *L. delicatula*

On 1 October, courtship behaviour was observed, where a male flapped its wings around a female (Movie S1). Mating occurred in the reverse mating position (Movie S2). Oviposition was observed on various plants, including *A. altissima*, and on building walls (Fig. S2f). As a behaviour associated with oviposition, *L. delicatula* laid eggs with white substances on the walls (Movie S3) or plants. Adult females repeated this oviposition behaviour while moving toward the head or tail side, laid 1–12 eggs, and returned to the start position. They then moved sideways of the start position and repeated the oviposition behaviour. After laying eggs, the adult female released a white, viscous liquid substance from the tail and evenly spread it over the egg mass (Movie S4). The white substance changed over time and settled as a brown-grey waxy substance after two hours (Fig. S2f).

### Survey of egg mass and influence of wax-removal from egg masses

Almost all egg masses were covered with the waxy substance (99/100 egg masses). The number of eggs per egg mass (n = 43) was 40.35 ± 5.31 (mean ± SD).

**Figure 3:**
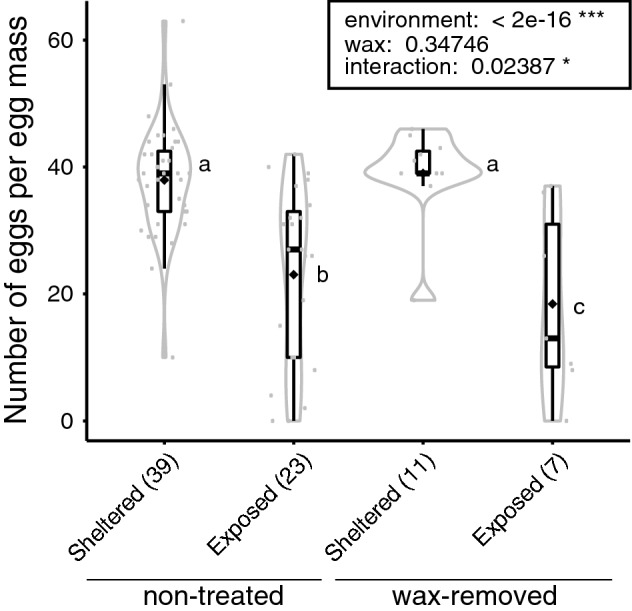
Influence of wax-coating and shelter on the number of eggs. Violin plots with overlaid box plots represent the distribution of the remaining number of eggs. The solid diamonds indicate the average values. Each grey dot indicates the number of eggs per egg mass. Sample size is provided in parenthesis. The results of two-way Analysis of Variance (ANOVA) using Generalized linear Modelling (GLM) with a Poisson error structure are shown in the box (*P < 0.05, ***P < 0.001). Different letters (a, b and c) indicate statistically significant differences (Tukey’s test, P < 0.05).

On 14 October, the number of eggs in the intact egg masses was 39.5 ± 5.57 (mean ± SD) in sheltered places and 39.0 ± 9.27 (mean ± SD) in exposed places, respectively. There was no significant difference in the number of eggs between the two place types (Wilcoxon rank sum test, P = 0.948). Figure 3 shows the number of eggs per egg mass two weeks after wax removal. At the time, the wax remained almost completely on the egg masses in the sheltered places, but the wax and some eggs were peeled off from egg masses probably by wind and rainfall action in the exposed places. Two-way Analysis of Variance (ANOVA) using Generalized Linear Modelling (GLM) revealed that the number of eggs was significantly lower in exposed places than in sheltered places (Fig. 3). Interaction between egg-deposit environment and wax-coating status was observed. In the egg masses formed in exposed places, the number of eggs in wax-removed egg masses was significantly lower than that in non-treated egg masses.

### Influences of egg-deposited site and wax-coating on the hatching rate

**Figure 4:**
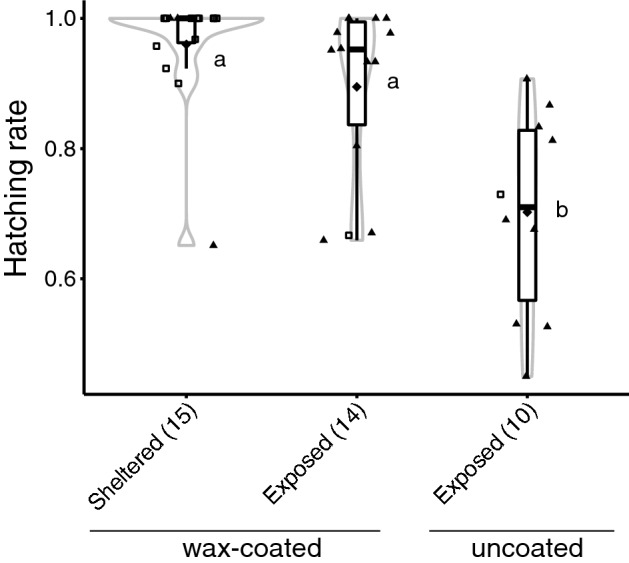
Hatching rate of eggs laid in different environments. Violin plots with overlaid box plots represent the distribution of hatching rate of the eggs. The solid diamonds indicate the average values. Open rectangles show eggs laid on building walls, while solid triangles show eggs laid on *Ailanthus altissima*. Sample size is provided in parenthesis. The different letters (a, b) indicate statistically significant differences (P < 0.05, Mann–Whitney U test after Bonferroni correction).

The state of wax coating of the overwintered egg masses was investigated in Midori. It was found that all the egg masses formed in sheltered places (most of them were in the buildings) were covered with waxy deposits (Fig. 4). In contrast, most of the egg masses formed in exposed places were found on *A. altissima*. Among these, 14 egg masses were covered with wax. However, the wax from the 10 egg masses had been partially or completely removed naturally. Wax-retaining egg masses deposited in exposed places tended to have a slightly lower hatching rate than those in sheltered areas, but no significant difference was detected. The dewaxed egg masses in the exposed area had significantly lower hatchability than those in the other two areas (Fig. 4).

### Molecular phylogenetic analyses

**Figure 5:**
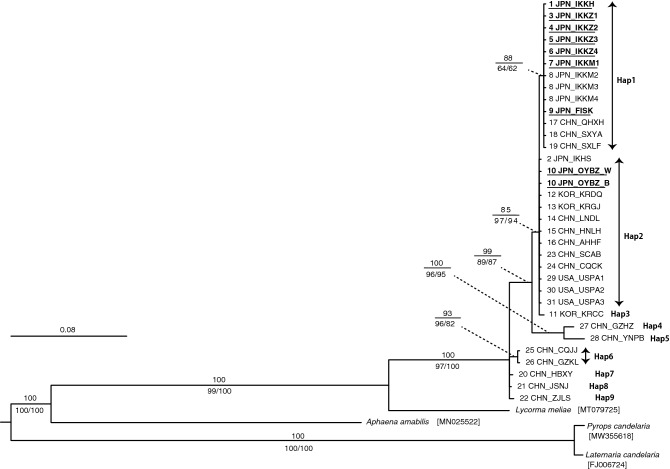
Molecular phylogenetic analysis of *Lycorma delicatula* based on integrated sequences of ND2 and ND6 genes. The topology and branch lengths shown were obtained by Bayesian inference methods. Scale bar indicates 0.08 substitutions per site. Posterior probabilities (PP) are shown above branches to indicate the level of support for each node. Bootstrap values from maximum-likelihood (ML) and maximum parsimony (MP) analyses are shown below the nodes (only values ≥ 50% are shown), respectively. Sequences obtained in this study are underlined and in bold type. Numbers at the head of the sequences correspond to the site numbers in Fig. S1 and Table S1. Labels of the sequences correspond to the codes in Table S1. Haplotypes are shown on the right in bold type. Sequence accession numbers of outgroup species are in brackets.

Our analyses revealed nine different haplotypes in the samples (Fig. 5). Almost all the samples in the Hokuriku region (Fig. S1b) belonged to haplotype 1, which included samples from the northwestern area of China (Fig. 5, Fig. S1 and Table S1). One sample (JPN_IKHS) collected from Hokuriku in 2010^11^ and two samples from Bizen City that included both white and blue-green hindwing individuals belonged to haplotype 2, which included the samples from central China, Korea, and the USA (Fig. 5 and Fig. S1).

## Discussion

Surveys at the two sites in Kanazawa City showed that the 1st instar larvae had hatched by June 2020 (Fig. 1). After 1 June, the population age structure changed every two weeks until the emergence of 4th instar larvae, which were numerous on 15 and 28 July. Adults were mainly detected on 12 August. This suggests that *L. delicatula* has a univoltine life cycle in this region, as reported in South Korea^22^ and Pennsylvania, USA^3^. The results also indicate that the 1st to 3rd instar larvae molt approximately every two weeks, and the period of development from the 4th instar to the adult phase is approximately one month in this region.

The patterns in which adults were captured differed significantly between females and males (Fig. 2). In August, a larger number of females were captured than males. After mid-September, when breeding began, the numbers of females and males captured were approximately equal. Our survey revealed that all individuals had reached adulthood by late August (Fig. 1). Hence, it is unlikely that males emerged much later than females, at least in the survey area up to three meters above the ground. Domingue et al*.*^23^ reported a similar female bias just after adult emergence based on a survey of large *A. altissima* trees up to four meters above the ground. They reported all-female aggregations on the trunks and exposed roots of larger *A. altissima* trees in the same period as that observed in this study (Fig. S2c,d). Female aggregation is suggested to be a behavior that causes them to crowd into a limited area to feed on optimal resources for producing viable egg masses^23^. It has also been reported that a high proportion of males are distributed on smaller trees of *A. altissima*, *Vitis* sp., and other plant species; however, the number of *L. delicatula* males on such plants are remarkably lower than those on the larger *A. altissima*^23^. Therefore, it is not fully understood why there were fewer males during the early adult emergence period in the survey areas. It is possible that males are distributed in higher positions of the host trees in the early stage of adult development. During the breeding season, courtship behaviour by males (Movie S1) and mating (Movie S2) were frequently observed in the survey area, as previously reported^24^. Males might change their distribution to nearer ground level during these periods. To clarify this, it will be necessary to expand the survey area to the upper parts of trees in the future.

*Lycorma delicatula* is known to be polyphagous but feeds mainly on *A. altissima*^1,3,4,8,25^. In the present study, most *L. delicatula* were observed on *A. altissima* (Fig. S2a–d), although some individuals were also observed on wild grapevine *A. glandulosa* var. *heterophylla* (Fig. S2e). Wild grapevine is also a favourite host plant of *L. delicatula*, as previously reported^3,8,26^. In addition to the host plants, many egg masses were laid on non–plant materials such as building walls (Fig. S2f), as reported previously^3,4,8,27^.

This study showed that most of the eggs of *L. delicatula* were covered with waxy deposits (99/100 egg masses), as reported previously^3,8^. The role of wax in *L. delicatula* is thought to protect eggs from environmental and biotic factors such as natural enemies^14,28^. In this study, we obtained data supporting the possibility that wax functions against some environmental factors. We observed a significant decrease in the number of eggs per egg mass in exposed environments compared to that in sheltered environments due to peeling off, likely a result of wind and rainfall action. When the wax was removed, the egg numbers per egg mass decreased further (Fig. 3). Moreover, this study showed that the hatching rate of overwintered eggs was significantly reduced when the wax was removed from the egg mass that formed in exposed places (Fig. 4). These results suggest that egg survival is greatly affected by environmental factors, such as wind and rainfall, and that wax may play a role in protecting eggs from these factors. To clarify this, a more detailed analysis should be conducted in an environment where the amount and intensity of wind and rainfall are strictly controlled.

To determine the genetic structure of *L. delicatula* populations in Japan, we conducted a phylogenetic analysis using ND2 and ND6 gene sequences for the samples collected from nine sites in the Hokuriku region and one site in the Okayama Prefecture (Fig. S1a,b, and Table S1). The occurrence of *L. delicatula* was recently confirmed from Okayama^18^; in this population, in addition to individuals with white hindwings, many individuals with blue-green coloured hindwings^28^ have also been reported^18^. In our analysis, we included both colour types collected from Okayama, and the gene sequence data obtained in previous studies^11,21^. The results showed that all the samples were classified into one of nine different lineages (i.e. haplotypes), whose geographic distributions were almost consistent with the results of the previous study by Du et al*.*^21^. All samples collected from the Hokuriku region (Fig. S1b) in Japan, except for that from Hakusan (JPN_IKHS), had identical sequences and belonged to the same clade as samples from the northwestern area of China (Fig. 5 and Fig. S1a). However, both hindwing colour variations (white and blue-green) from Okayama had identical sequences, and belonged to the same haplotype as the samples from the central area of China, South Korea, and the USA (Fig. 5 and Fig. S1). These results indicate that the genetic structure of *L. delicatula* in Japan is divided into at least two groups and supports that each group has a history of invasion and colonisation from different regions. Interestingly, this study revealed that the sample collected from Hakusan in Japan in 2010 (site no. 2 in Fig. S1b and Table S1) belonged to the same haplotype as the samples from the central areas of China, South Korea, and the USA, but not to those collected from the same Hokuriku region in Japan in 2020 (Fig. 5 and Fig. S1b). This may indicate that in the last decade, the central China haplotype previously existing in the Hokuriku area has been replaced by the northwestern China haplotype. To clarify this, a more detailed analysis using high-resolution markers^7,21,29^ and a larger sample size, including old, preserved specimens that were captured during the first invasion into the Hokuriku area, is required.

*Lycorma delicatula* has rapidly expanded its distribution in several countries. In South Korea, the first specimen-confirmed report of *L. delicatula* was published in 2004. Thereafter, its distribution expanded throughout South Korea, and population densities increased by 2011^4,8^. In the USA, it was first detected in Pennsylvania in 2014^9^, and by 2021, had expanded its distribution into 12 other surrounding states^4,10^ (Fig. S1c). In contrast, in Japan, the distribution of *L. delicatula* has been limited to the Hokuriku region (Fig. S1b) since it was first reported in the Ishikawa Prefecture in 2009^14^ until it was detected in Osaka Prefecture in 2017^17^, even though the preferred host plant, *A. altissima*, is distributed throughout Japan^19,20^. Various biotic and/or abiotic factors seem to be involved in this relatively slow expansion of distribution in Hokuriku, Japan. The most likely factor is the influence of climate, as shown previously^22,30,31^. Hokuriku has a large amount of precipitation, including snowfall in winter. For example, mean annual precipitation in Kanazawa is 2401.5 mm^32^, much higher than that of Philadelphia (1060.0 mm), and Seoul (1460.0 mm)^33^. Precipitation appears to cause a decrease in egg viability (Figs. 3 and 4). This might explain the suppressed distributional range expansion of *L. delicatula* from Hokuriku, although it would be necessary to confirm that egg mortality in the Hokuriku region is higher than in other regions in future studies. In addition, indigenous predators and parasitoids in the region may play an important role in suppressing the population of *L. delicatula*, which should also be explored in future research.

In Japan, *L. delicatula* has recently been found in Osaka^17^ and Okayama^18^, which are warm regions with relatively low-precipitation (mean annual precipitation in these areas are 1338.3 mm and 1143.1 mm, respectively^32^). The Okayama population has the same haplotype as the one that has rapidly increased in South Korea and the USA (Fig. 5 and Fig. S1). This may mean that the southwestern region of Japan is at high risk of *L. delicatula* invasion. Hence, detailed monitoring of *L. delicatula* is needed in these regions. Simultaneous preventative action to control the spread of *L. delicatula* is also required. Control using pesticides may adversely affect the indigenous species, therefore alternative methods should be used. Further verification on the vulnerability of dewaxed eggs of *L. delicatula* to precipitation (Figs. 3 and 4) is needed, but this study has provided valuable insights into how this pest insect could be managed in an environmentally friendly way. A deeper understanding of the specific ecology of invasive alien species is necessary for sustainable environmental conservation.

## Materials and methods

### Occurrence of the developmental stages in natural populations

Surveys were performed from June to November 2020 at two locations, Midori and Marunouchi, in Kanazawa City (Locality no. 4 and 5 in Fig. S1b and Table S1), Ishikawa Prefecture, Japan, approximately every 2 weeks. The two locations were approximately 6.5 km apart and the survey areas of the sites were ca. 10,000 m^2^ and 5000 m^2^, respectively. Almost all *L. delicatula* were found on *Ailanthus altissima* plants at these sites (Fig. S2a–d), but some were also found on *Ampelopsis glandulosa* var. *heterophylla* (Fig. S2e). Insects were collected with a sweep net at heights up to three meters from the ground. The number of insects collected and their developmental stages were recorded. Developmental stages and sex of the insects were identified by their size and morphological features, according to Dara et al*.*^3^. Sex was only identified in adults, because the sexes are indistinguishable at the immature stages.

### Mating and oviposition behaviours of *L. delicatula*

On 1 October, 2020, mating and oviposition behaviours were observed in Midori and Marunouchi. Both behaviours were recorded using a digital camera (Olympus Stylus TG-3 Tough). The videos were edited using the FFmpeg software (https://www.ffmpeg.org).

### Egg masses of *L. delicatula*

Egg mass surveys were conducted on October 1 and 14, 2020, in Midori. The presence or absence of a brown-grey wax coating on the egg surface was checked and recorded using 100 randomly chosen egg masses. To count the number of eggs in each mass, the waxes on 43 egg masses were removed with a paint brush, and images were taken using a Stylus TG-3 Tough (Olympus, Tokyo, Japan) digital camera. The total number of eggs in the egg mass was counted from the images.

### Wax removal from the egg surface

An investigation was conducted in Midori using egg masses that had been laid on building walls up to two meters above the ground (Fig. S2f). Fifty intact egg masses that had formed on sheltered walls (roofed) and 30 intact egg masses formed on exposed (unroofed) walls were randomly chosen. On 14 October 2020, waxy deposits were removed from the surfaces of some egg masses using a paint brush, and images were taken with an Olympus Stylus TG-3 tough digital camera. Two weeks later, waxes were removed from the remaining egg masses in both sheltered and exposed places. Images of all 80 wax-removed egg masses were obtained using the same equipment. The total number of eggs per egg mass was counted from the images.

### Influences of egg-deposit site and wax-coating on the hatching rate

To determine the hatching rate of overwintered eggs, a survey was conducted on 28 April, 2021, at Midori. In total, 39 egg masses were examined to determine the environmental conditions in which the egg mass was formed (i.e., sheltered or exposed place on the building walls or trees) and wax coating state (i.e., the whole egg mass was covered or uncovered). After the waxy deposits were removed from the egg masses using a paint brush, images were taken with an Olympus Stylus TG-3 tough digital camera; then, the eggs with opened operculums were considered to have successfully hatched. Egg masses containing unhatched eggs were carefully collected and moved to the laboratory where the temperature was kept at 25 °C. Egg hatching was observed for one month. The hatching rate of an egg mass was calculated as the number of successfully hatched individuals from the total egg number in each egg mass.

### Molecular phylogenetic analyses

*Lycorma delicatula* samples were collected from eight locations in Japan and one location in China (Fig. S1a,b, and Table S1). All specimens had white bands on the hindwings, except for one from Bizen City, which had a blue-green band^18^. The collected samples were immediately preserved in 100% acetone until DNA extraction^34^. DNA was extracted from the abdomen of an adult female using a conventional phenol extraction method. The purified DNA was resuspended in 200 µL of TE buffer (10 mM Tris–HCl [pH 8.0], 0.1 mM EDTA). The partial sequences of NADH dehydrogenase subunit 2 (ND2) and NADH dehydrogenase subunit 6 (ND6) were amplified with KOD-plus-ver.2 DNA polymerase (TOYOBO, Osaka, Japan). ND2 and ND6 were amplified using the primers Ld_ND2_238F and LD2_866R, and Ld_ND6_87F and Ld_ND6_480R, respectively, as described by Kim et al*.*^11^. The PCR temperature profile was 94 °C for 2 min, followed by 35 cycles of 94 °C for 15 s, 55 ℃ for 30 s, and a final extension at 68 °C for 1 min. The PCR products were purified using polyethylene glycol solution (PEG6000 20% and 2.5 mM). The PCR amplicons were directly sequenced in both directions with the same primers using the BigDye Terminator v3.1 Cycle Sequencing Kit and the Applied Biosystems 3500 Genetic Analyzer (Thermo Fisher Scientific, MA, USA). The sequences obtained were assembled and analysed using ATGC ver. 4 software (GENETYX, Tokyo, Japan).

The sequences were aligned using CLUSTAL W with MEGA X^36^. ND2 and ND6 sequences were integrated, and a total of 889 positions were included in the final dataset (gaps and stop codons were not included). This analysis involved published sequences of *L. delicatula* from previous studies^11,21^, and the phylogenetically closely related taxa *Lycorma meliae* (Accession No: MN025522), *Pyrops candelaria* (MW355618), and *Laternaria candelaria* (FJ006724) were used as outgroups. We performed phylogenetic analyses using Bayesian inference (BI), maximum-likelihood (ML), and maximum parsimony (MP) methods. In the BI analysis, the most appropriate model of sequence evolution (HKY + G model) was selected using the MEGA X model selection option. The parameters for the selected substitution model were estimated from the data. In total, 100,000 trees were obtained (ngen = 10,000,000, sample freq = 100) using MrBayes 3.2.7^35^, and the first 25% of these (25,000) were considered as the ‘burn in’ and discarded. A consensus tree of the remaining trees based on the 50% majority-rule was produced. Two independent runs were performed using the same model of sequence evolution. In the ML analysis, bootstrap analysis of 1000 replications was performed based on the same model as BI in MEGA X. Initial trees for the heuristic search were obtained automatically by applying Neighbor-Join and BioNJ algorithms to a matrix of pairwise distances estimated using the maximum composite likelihood approach. In the MP analysis, all characters were included and weighted equally, and 1000 bootstrap replicates were performed using MEGA X^36^. The Subtree-Pruning-Regrafting algorithm with search level 1, where the initial trees were obtained by the random addition of sequences (10 replicates), was used. The haplotypes were identified using the software of DnaSP 6.0^37^.

### Nucleotide sequence accession numbers

All sequences determined in this study have been submitted to the DDBJ/EMBL/GenBank database under the following accession numbers: LC649256–LC649265 and LC649266–LC649275 for ND2 and ND6, respectively (Table S1).

### Statistics

Two-sided binomial test with Bonferroni correction was applied to determine whether the proportion of adult *L. delicatula* significantly differed from the 1:1 sex ratio. The Mann–Whitney U test was used to evaluate the difference in the number of eggs included in an intact egg mass between the sheltered and exposed places. Two-way ANOVA using GLM with a Poisson error structure was used to assess the effect of egg-deposit environment (sheltered or exposed place), wax-coating status (wax coating or wax removed), and their interaction on the number of eggs per egg masses. Tukey’s test was used for its post-hoc multiple comparisons. The Mann–Whitney U test, after Bonferroni correction, was used to evaluate differences in hatching rates. All statistical analyses were conducted using R software^38^ v. 3. 3. 3.

## Supplementary Information

Supplementary Figure S1.

Supplementary Figure S2.

Supplementary Video S1.

Supplementary Video S2.

Supplementary Video S3.

Supplementary Video S4.

Supplementary Table S1.

## Data Availability

This article has no additional data.
