# Supplementary material for: Ecology and genetic structure of the invasive spotted lanternfly Lycorma delicatula in Japan where its distribution is slowly expanding
Source: Sci Rep. 2022 Feb 1;12:1543. doi: 10.1038/s41598-022-05541-z (PMC8807778; doi:10.1038/s41598-022-05541-z)
Supplement: Supplementary file 1 — Supplementary Figure S1. [file 41598_2022_5541_MOESM1_ESM.pdf]

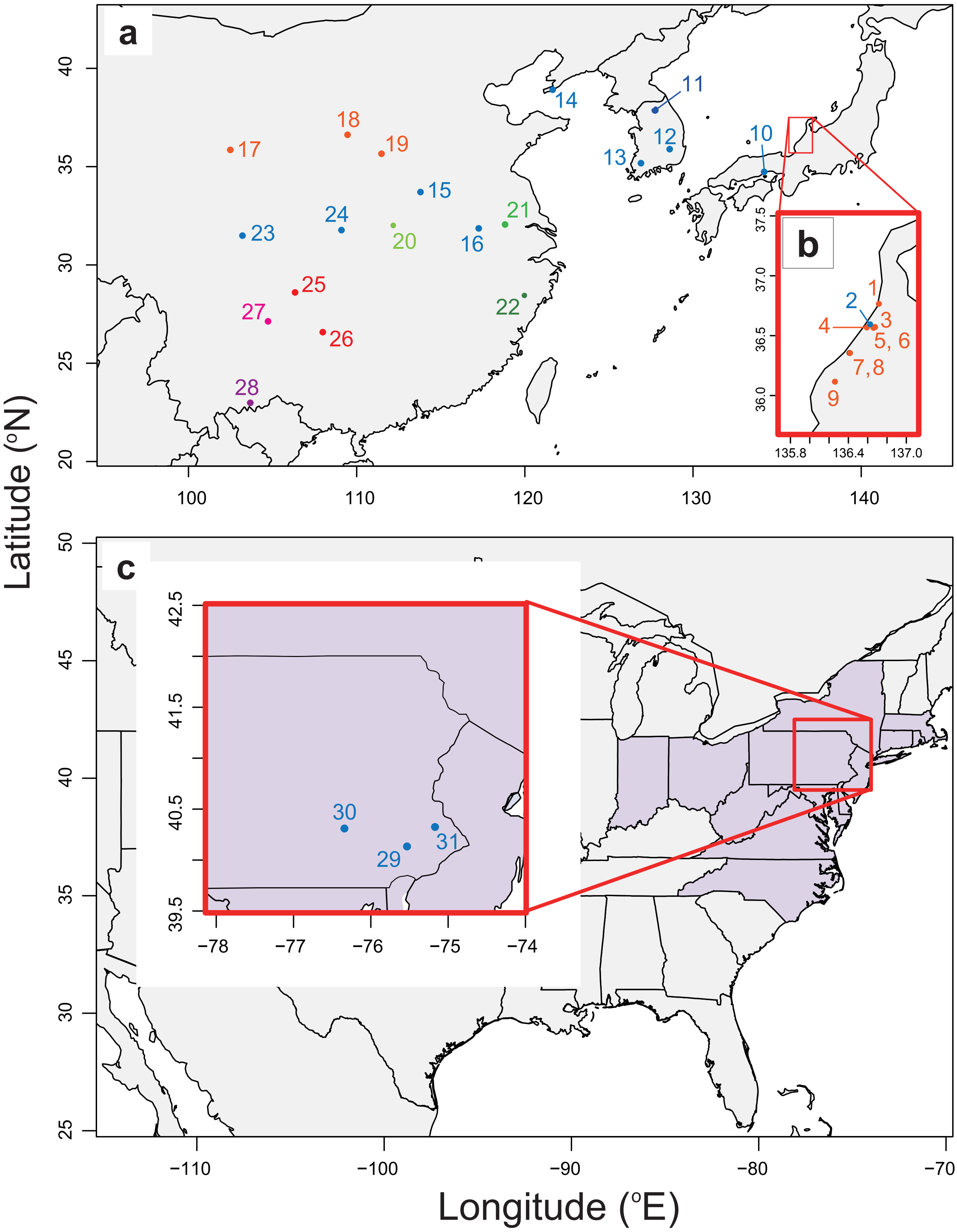

Fig. S1 Geographic distribution of the sampling sites in East Asia (a), Hokuriku region in Japan (b), and the USA (c). The numbers correspond to the numbers in Fig. 5 and Table S1. The colors of the sites show the difference in the mitochondrial haplotypes of the individuals detected from the sites, which corresponds to the result of Fig. 5. In (c), the states colored in purple are the locations where the distribution of *L. delicatula* was confirmed (NYSIPM: https://nysipm.cornell.edu/environment/invasive-species-exotic-pests/spotted-lanternfly/).
