# Supplementary material for: Ecology and genetic structure of the invasive spotted lanternfly Lycorma delicatula in Japan where its distribution is slowly expanding
Source: Sci Rep. 2022 Feb 1;12:1543. doi: 10.1038/s41598-022-05541-z (PMC8807778; doi:10.1038/s41598-022-05541-z)
Supplement: Supplementary file 2 — Supplementary Figure S2. [file 41598_2022_5541_MOESM2_ESM.pdf]

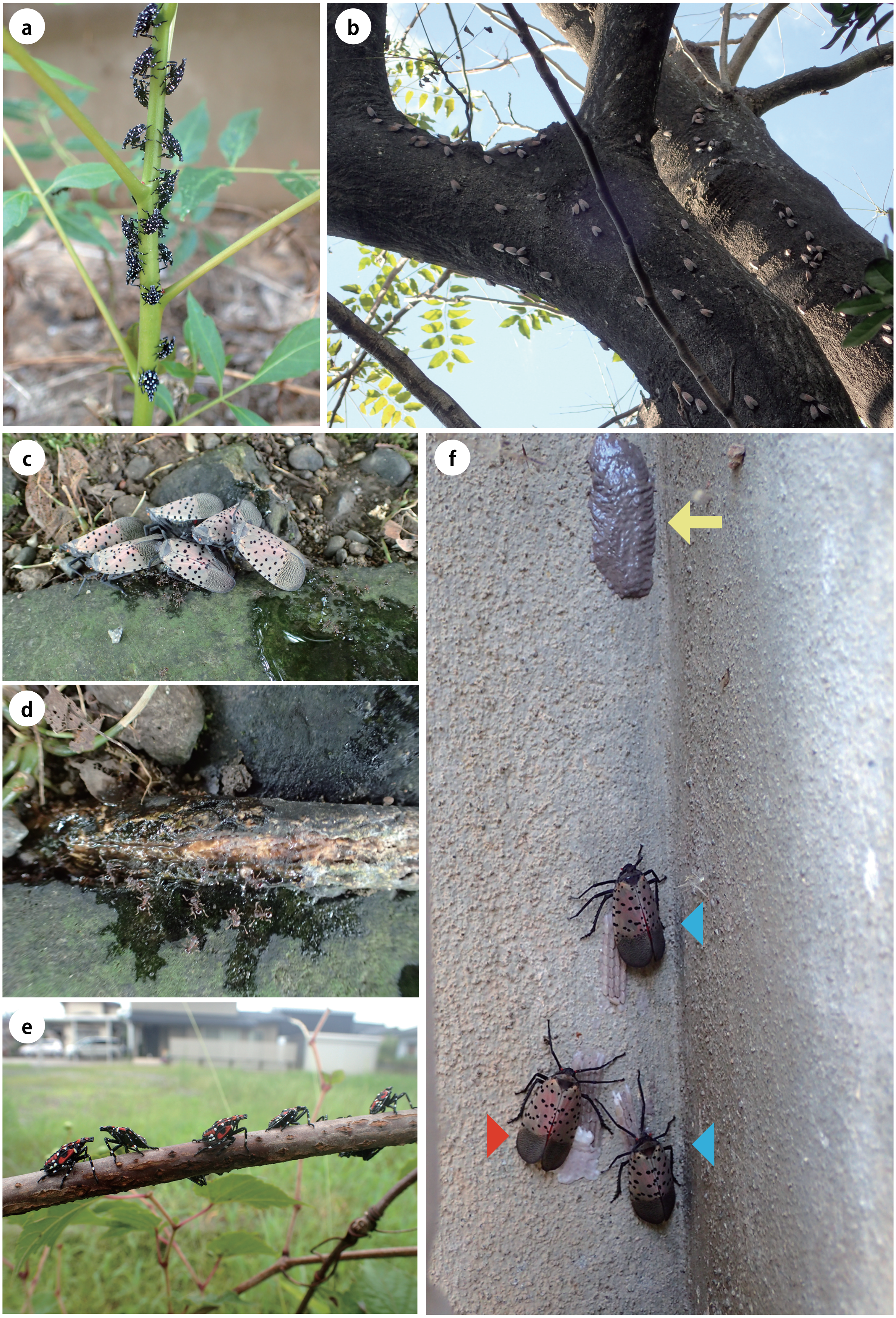

Fig.S2 *Lycorma delicatula* on the host plants and building walls. (a) Young nymphs on the seedling of *A. altissima*. (b) Adults on *A. altissima*. (c) Adult females feeding on the root of *A. altissima*. (d) The root of *A. altissima* after the females were removed. (e) The 4th instar nymphs on *A. glandulosa var. heterophylla*. (f) Adult females and egg masses. Blue arrowhead, female reproducing eggs; Red arrowhead, female releasing white waxy deposit over the egg mass; Yellow arrow, an egg mass covered with brown-grey wax.
