# Supplementary material for: Ecology and genetic structure of the invasive spotted lanternfly Lycorma delicatula in Japan where its distribution is slowly expanding
Source: Sci Rep. 2022 Feb 1;12:1543. doi: 10.1038/s41598-022-05541-z (PMC8807778; doi:10.1038/s41598-022-05541-z)
Supplement: Supplementary file 7 — Supplementary Table S1. [file 41598_2022_5541_MOESM7_ESM.pdf]

Table S1. Samples of *L. delicatula* in this study

Locality no.[†]	Sample locality	Sample code[‡]	Latitude	Longitude	Sampling date	Accession number (ND2 / ND6)	Reference
	Japan						
1	Takamatsu, Kahoku, Ishikawa	JPN_IKKH	36.7659	136.7156	2020-08	LC649256 / LC649266	This study
2	Hakusan, Ishikawa	JPN_IKHS	36.5946	136.6255	2010-09	KC422370 / KC422388	Kim *et al*. 2013
3	Utatsuyama, Kanazawa, Ishikawa	JPN_IKKZ1	36.5722	136.6762	2019-10	LC649257 / LC649267	This study
4	Midori, Kanazawa, Ishikawa	JPN_IKKZ2	36.5688	136.5907	2020-08	LC649258 / LC649268	This study
5	Marunouchi, Kanazawa, Ishikawa	JPN_IKKZ3	36.5687	136.6585	2019-11	LC649259 / LC649269	This study
6	Marunouchi, Kanazawa, Ishikawa	JPN_IKKZ4	36.5656	136.6600	2020-08	LC649260 / LC649270	This study
7	Shichomachi, Komatsu, Ishikawa	JPN_IKKM1	36.3555	136.4132	2019-10	LC649261 / LC649271	This study
8	Futsumachi, Komatsu, Ishikawa	JPN_IKKM2	36.3551	136.4172	2017-09	MT079463[¶]	Du *et al*. 2021
8	Futsumachi, Komatsu, Ishikawa	JPN_IKKM3	36.3551	136.4172	2017-09	MT079464[¶]	Du *et al*. 2021
8	Futsumachi, Komatsu, Ishikawa	JPN_IKKM4	36.3551	136.4172	2017-09	MT079465[¶]	Du *et al*. 2021
9	Maruokacho, Sakai, Fukui	JPN_FISK	36.1156	136.2608	2019-10	LC649262 / LC649272	This study
10	Honami, Bizen, Okayama	JPN_OYBZ_W[§]	34.7348	134.2293	2020-08	LC649263 / LC649273	This study
10	Honami, Bizen, Okayama	JPN_OYBZ_B[§]	34.7348	134.2293	2020-08	LC649264 / LC649274	This study
	South Korea						
11	Seoksa-dong, Chuncheon, Gangwon-do	KOR_KRCC	37.8695	127.7423	2018-08	MT079523[¶]	Du *et al*. 2021
12	Daehyeon-dong, Buk-gu, Daegu	KOR_KRDQ	35.8901	128.6113	2018-08	MT079543[¶]	Du *et al*. 2021
13	Yongbong-dong, Buk-gu, Kwangju	KOR_KRGJ	35.1761	126.9036	2018-08	MT079553[¶]	Du *et al*. 2021
	China						
14	Zhongshan District, Dalian, Liaoning	CHN_LNDL	38.9109	121.6529	2017-08	MT079593[¶]	Du *et al*. 2021
15	Yancheng District, Luohe, Henan	CHN_HNLH	33.7066	113.7939	2017-09	MT079443[¶]	Du *et al*. 2021
16	Shushan District, Hefei, Anhui	CHN_AHHF	31.8602	117.2574	2020-10	LC649265 / LC649275	This study
17	Xunhua County, Haidong, Qinghai	CHN_QHXH	35.8514	102.4873	2017-07	MT079613[¶]	Du *et al*. 2021
18	Baota District, Yan'an, Shannxi	CHN_SXYA	36.6190	109.4571	2017-07	MT079683[¶]	Du *et al*. 2021
19	Qvwo Country, Linfen, Shanxi	CHN_SXLF	35.6569	111.4757	2018-07	MT079663[¶]	Du *et al*. 2021
20	Xiangcheng District, Xiangyang, Hubei	CHN_HBXY	32.0106	112.1743	2017-08	MT079433[¶]	Du *et al*. 2021
21	Xuanwu District, Nanjing, Jiangsu	CHN_JSNJ	32.0559	118.8242	2017-08	MT079473[¶]	Du *et al*. 2021
22	Liandu District, Lishui, Zhejiang	CHN_ZJLS	28.4473	119.9728	2017-08	MT079715[¶]	Du *et al*. 2021
23	Li Country, Aba, Sichuan	CHN_SCAB	31.4903	103.2094	2017-09	MT079623[¶]	Du *et al*. 2021
24	Chengkou District, Chongqing	CHN_CQCK	31.7711	109.0956	2017-08	MT079363[¶]	Du *et al*. 2021
25	Jiangjin District, Chongqing	CHN_CQJJ	28.6017	106.3381	2017-08	MT079373[¶]	Du *et al*. 2021
26	Kaili City, Qiandongnan, Guizhou	CHN_GZKL	26.5808	107.9831	2017-09	MT079403[¶]	Du *et al*. 2021
27	Hezhang Country, Bijie, Guizhou	CHN_GZHZ	27.1307	104.7313	2017-08	MT079393[¶]	Du *et al*. 2021
28	Pingbian Country, Honghe, Yunnan	CHN_YNPB	22.9863	103.6751	2017-08	MT079713[¶]	Du *et al*. 2021
	USA						
29	647 Nutt Road, Chester, Pennsylvania State	USA_USPA1	40.1336	−75.5319	2017-08	MT079707[¶]	Du *et al*. 2021
30	2208 S 5th Ave, Lebanon, Pennsylvania State	USA_USPA2	40.3098	−76.3416	2017-09	MT079708[¶]	Du *et al*. 2021
31	Peace Valley Park, Bucks, Pennsylvania State	USA_USPA3	40.3246	−75.1697	2017-09	MT079709[¶]	Du *et al*. 2021

[†] The population numbers correspond to the numbers shown in Fig. 5 and Fig. S1.

[‡] Code is the abbreviation of each location and the numbers indicate different populations in the same location. JPN; Japan, KOR; Korea, CHN; China, USA; United States of America. The Code corresponds to the sample label in molecular phylogenetic analysis shown in Fig. 5.

[§] JPN_OYBZ_W is the sample with white bands in hindwings. JPN_OYBZ_B is the sample with blue bands in hindwings.

[¶] Whole mitochondria genome sequence
